# Risk prediction for early-onset gastric carcinoma: a case-control study of polygenic gastric cancer in Han Chinese with hereditary background

**DOI:** 10.18632/oncotarget.9025

**Published:** 2016-04-26

**Authors:** Jiajia Yuan, Yanyan Li, Tiantian Tian, Na Li, Yan Zhu, Jianling Zou, Jing Gao, Lin Shen

**Affiliations:** ^1^ Department of Gastrointestinal Oncology, Key laboratory of Carcinogenesis and Translational Research (Ministry of Education/Beijing), Peking University Cancer Hospital and Institute, Beijing, China

**Keywords:** gastric cancer, susceptibility, polymorphisms, risk classifications, Pathology Section

## Abstract

Recent genomewide studies have identified several germline variations associated with gastric cancer. The aim of the present study was to identify, in a Chinese Han population, the individual and combined effects of those single nucleotide polymorphisms (SNPs) that increase the risk of early-onset gastric cancer. We conducted a case-control study comprising 116 patients with gastric cancer as well as 102 sex- and age-matched controls and confirmed that the SNPs *MUC1* (mucin 1) rs9841504 and *ZBTB20* (zinc finger and BTB domain containing 20) rs4072037 were associated with an increased gastric cancer risk. Of the 116 patients diagnosed with cancer, 65 had at least 1 direct lineal relative with carcinoma of the digestive system or breast/ovarian cancer. These 65 had another 4 SNPs associated with gastric cancer susceptibility: *PSCA* (prostate stem cell antigen) rs2294008, *PLCE1* (*phospholipase C epsilon 1*) rs2274223, *PTGER4*/*PRKAA1* (prostaglandin E receptor 4/protein kinase AMP-activated catalytic subunit alpha 1) rs13361707, and *TYMS* (thymidylate synthetase) rs2790. However, each of these low-penetrance susceptibility polymorphisms alone is not considered influential enough to predict the absolute risk of early-onset gastric cancer. Thus we decided to study different combinations of polygenes as they affected for our population. Those subjects with both the risk alleles *MUC1* rs9841504 and *ZBTB20* rs4072037 had a greater than 3-fold increased risk of gastric cancer. Also those with a hereditary background including the risk alleles *PLCE1* rs2274223 and *PTGER4*/*PRKAA1* rs13361707 were 3 times more susceptible to cardia cancer than those without. These findings show that the study of combined polymorphisms, instead of single low-penetrance variations in susceptibility, may lead to a high-risk classification for a specific population.

## INTRODUCTION

Gastric cancer is the fourth most common cancer worldwide and the second most common cause of death due to cancer globally.[1] Approximately 10% of all cancer deaths worldwide are gastric cancer−specific, with 40% occurring in China.[2] Owing to improved living standards and the eradication of *Helicobacter pylori*, especially in East Asia and Latin America,[3] the incidence of gastric cancer has declined in most parts of the world since the 1900s.[4, 5] Since 1970s, however, the overall incidence of noncardia gastric cancer in a subgroup of the white population between 25 and 39 years of age has increased by two thirds.[6] Similar results have been observed in China, where the incidence in a subgroup of the rural population between 15 and 44 years of age has increased since the 1990s.[7] There are several reasons for this paradoxical phenomenon. Although factors like *H. pylori* have recently been better recognized and understood, other environmental factors, such as air pollution and climate change, can induce effects that seem relatively small but that accumulate yearly and thus affect these specific generations.[8, 9] Also, after the decline of *H. pylori*i infection, other risk factors—such as Epstein-Barr virus, which was unmasked by the eradication of *H. pylori*—can also increase the risk of carcinogenesis.[6] Third, the accumulated genetic variations in carcinogenesis have now become more marked, leading to an earlier onset disease.[10, 11]

Genetic variations in breast cancer (e.g., *BRCA1* and *BRCA2*) are highly penetrant, suggesting a strong linkage with family history and genetic susceptibility.[12] Similarly, a relationship between germline alterations in *CDH1* (E-cadherin) and hereditary diffuse gastric cancer with family clustering has been observed in western countries [13]; however, rare families have also been reported in Asian countries.[14–16] Finally, in sporadic gastric cancer, genetic susceptibility to the SNP *CDH1* rs16260 was reported at odds ratios of 1.20 in European and 0.93 in Asian populations.[17, 18] We undertook further study of genetic-related gastric cancer in an Asian population, much as in the Genome-Wide Association Studies (GWAS). The latter identified several risk-associated loci with genetic susceptibility, including the SNPs *PSCA* rs2976392 (strong linkage disequilibrium with rs2294008), *PLCE1* rs2274223, ZBTB20 rs9841504, and *PTGER4/PRKAA1*rs13361707.[19–21] *MUC1* rs9841504 and *TYMS* rs2790 have been recognized as risk alleles in similar studies of gastric cancer.[25, 26] However, results have not always been consistent, possibly owing to varying hereditary traits.[22–25]

Polygenic approaches have been attempted to predict and prevent breast and bladder cancers stemming from low-penetrance mutations.[27, 28] Recently several genetic susceptibility loci associated with gastric cancer risk have been identified and verified, and it was suggested that “sporadic” cancer be called “polygenic” instead of “nonhereditary.”[29] Although twin studies have suggested that many ‘sporadic’ cancers show little or no heritability, Lu et. al. have demonstrated that several ‘sporadic’ cancers have a significant inherited component.[29] We named them as ‘hereditary background’ in this paper. In our research involving Chinese Han individuals of age 50 years or below with a hereditary background of malignancy, we were able to identify a number of potential risk alleles in polygenic gastric cancer. The primary purpose of our study was to elucidate the combined effect of such early-onset risk alleles.

## RESULTS

### Characteristics of study subjects

**Figure 1:**
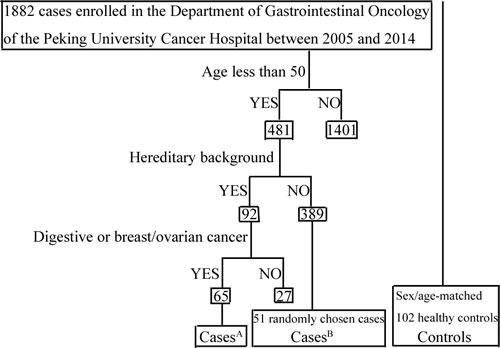
Flowchart of cases selection: A brief description of the patient-selection procedures

This study included 116 Chinese Han individuals less than 50 years of age with gastric cancer and 102 healthy sex- and age-matched controls. All were retrospectively chosen between March 2005 and June 2014 from the Department of Gastrointestinal Oncology of the Peking University Cancer Hospital (Table 1). Sixty-five individuals in our study who had already been diagnosed with cancer had at least 1 direct lineal relative with carcinoma of the digestive system or breast/ovarian cancer; therefore these subjects were assumed to have a hereditary background of malignancy (Figure 1).

**Table 1:** Characteristics of patients and controls

	Cases^A^* (%)	Cases^B^* (%)	Total Cases (%)	Controls (%)
Total number	*N* = 65	*N* = 51	*N* = 116	*N* = 102
Mean age, years	40.4	41.5	40.8	39.9
Age				
≤40	38.5	35.3	37.1	43.1
>40 and <50	61.5	64.7	62.9	56.9
Sex				
Male	64.6	60.8	62.9	57.8
Female	35.4	39.2	37.1	42.2
Location				
Cardia	18.5	31.4	24.1	—
Noncardia	80.0	68.6	75.0	—
Unknown	1.5	—	0.9	—
Pathology				
Intestinal type	20.0	31.4	25.0	—
Diffuse type	40.0	51.0	44.8	—
Mixed type	13.9	15.7	14.7	—
Unknown	26.1	1.9	15.5	—

* Cases^A^ were patients with a cancer history in lineal kin (genetic background); cases^B^ were patients without a cancer history in lineal kin.

### The risk of individual loci for early-onset gastric carcinoma

We investigated SNPs of *MUC1* rs9841504, *ZBTB20* rs4072037, *PSCA* rs2294008, *PLCE1* rs2274223, *PTGER4/PRKAA1* rs13361707, and *TYMS* rs2790. The Hardy-Weinberg equation was used to compare the observed and expected genotype frequencies (Supplementary Table 1). The frequencies of these loci in the general population were similar to those found by the Human Genome Project (Supplementary Table 2). Compared with the low-risk allele, the high-risk allele of SNP rs4072037 in *MUC1*, with a frequency of 89% in the group of all gastric cancer cases under 50 years of age, had a per-allele risk of 1.76 (95% CI 1.01-3.05, *P* = 0.045*) adjusted for sex and age in an unconditional logistical model (Table 2). Similarly, SNP rs9841504 in *ZBTB20* had a per-allele risk of 2.21 (95% CI 1.20-4.05, *P* = 0.011*). However, for SNPs rs2294008, rs2274223, rs13361707, and rs2790, a more obvious difference was observed in a comparison between groups of gastric cancer patients with or without hereditary background in the allele-specific model (Table 2). Similar results were obtained in the codominant, dominant, and recessive models (Supplementary Table 3). According to the multiplicative polygenic model applied in breast cancer,[30] we calculated that all these 6 variants account for 32% of the genetic risk of gastric cancer (Supplementary Table 4; see Supplementary Materials for detail).

**Table 2:** Comparison of each SNP between Cases/Cases^A^ and controls or Cases^A^ and Cases^B^ in the per-allele model*

dbSNP No.	Gene	Cases*vs*. Controls			Cases^A^ *vs*. Controls			Cases^A^ *vs*. Cases^B^		
		OR†	95% CI	*P* value	OR†	95% CI	*P* value	OR†	95% CI	*P* value
rs4072037	*MUC1*	1.76	1.01–3.05	0.045*	1.78	0.92–3.46	0.088	1.59	0.58–4.35	0.367
rs9841504	*ZBTB20*	2.21	1.20–4.05	0.011*	2.25	1.14–4.43	0.019*	0.72	0.32–1.65	0.442
rs2294008	*PSCA*	1.33	0.88–2.01	0.171	1.74	1.09–2.77	0.021*	2.03	1.11–3.72	0.022*
rs2274223	*PLCE1*	1.13	0.71–1.79	0.604	1.55	0.93–2.58	0.096	2.41	1.14–5.11	0.021*
rs13361707	*PTGER4/PRKAA1*	1.05	0.72–1.53	0.807	1.2	0.77–1.88	0.421	1.62	0.91–2.90	0.102
rs2790	*TYMS*	1.23	0.83–1.82	0.302	1.43	0.91–2.24	0.124	1.57	0.88–2.81	0.129

* The per-allele model compares the difference between minor and major alleles.

† In the logistic regression model OR is adjusted for sex and age.

### Subgroup analysis

In our subgroup analysis, we divided those SNPs into particular groups according to the Lauren classification and tumor locations (Table 3, Supplementary Table 5). The SNPs rs9841504, rs2294008, and rs2790 increased the risk of noncardia gastric cancer, whereas rs2274223 increased the risk of cardia cancer. In contrast, rs4072037 increased the risk of diffuse-type gastric cancer, while rs2294008 increased the risk of intestinal-type gastric cancer. The age difference was not significant; however, rs9841504, rs2274223, and rs2790 increased the risk of gastric cancer in males.

**Table 3:** Subgroup analysis between Cases^A^ and Controls*

Exposure		*MUC1* rs4072037			*ZBTB20* rs9841504			*PSCA* rs2294008		
		OR	95% CI	*P* value	OR	95% CI	*P* value	OR	95% CI	*P* value
Sex†										
	Male	1.83	0.79–4.25	0.158	3.25	1.31–8.08	0.011*	1.69	0.91–3.14	0.096
	Female	1.61	0.54–4.84	0.393	1.15	0.38–3.51	0.805	1.77	0.85–3.70	0.127
Age‡										
	≤40	1.25	0.47–3.33	0.654	2.73	0.96–7.75	0.059	1.73	0.84–3.58	0.137
	40–50	2.29	0.91–5.76	0.077	1.79	0.72–4.44	0.208	1.72	0.92–3.21	0.090
Location										
	Noncardia	1.51	0.76–3.00	0.239	2.52	1.24–5.10	0.010*	1.86	1.13–3.07	0.015*
	Cardia	—	—	0.99	1.61	0.43–6.02	0.478	1.29	0.52–3.19	0.583
Pathology										
	Diffuse	5.29	1.22–22.88	0.026	1.58	0.61–4.14	0.348	1.45	0.76–2.79	0.263
	Intestinal	2.75	0.60–12.53	0.191	0.51	0.06–4.21	0.531	3.4	1.42–8.17	0.006†
	Mixed	1.14	0.31–4.21	0.849	3.65	1.02–13.10	0.047*	1.71	0.62–4.74	0.302

Exposure		*PLCE1* rs2274223			*PTGER4* and*PRKAA1* rs13361707			*TYMS* rs2790		
		OR	95% CI	*P* value	OR	95% CI	*P* value	OR	95% CI	*P* value
Sex†										
	Male	2.00	1.02–3.92	0.044*	1.25	0.71–2.21	0.441	1.96	1.09–3.53	0.025*
	Female	1.07	0.47–2.44	0.879	1.15	0.55–2.40	0.706	0.84	0.40–1.76	0.639
Age‡										
	≤40	1.46	0.65–3.27	0.363	1.01	0.50–2.04	0.968	1.31	0.64–2.65	0.460
	40–50	1.63	0.83–3.19	0.158	1.38	0.76–2.48	0.289	1.49	0.82–2.71	0.193
Location										
	Noncardia	1.46	0.84–2.54	0.180	1.09	0.67–1.76	0.731	1.58	0.97–2.56	0.065
	Cardia	2.35	0.96–5.75	0.062	2.03	0.82–4.99	0.125	1.14	0.47–2.74	0.769
Pathology										
	Diffuse	1.54	0.77–3.09	0.224	1.44	0.77–2.70	0.259	1.55	0.83–2.89	0.171
	Intestinal	1.94	0.75–5.02	0.171	1.44	0.61–3.38	0.405	1.36	0.57–3.29	0.489
	Mixed	0.54	0.12–2.49	0.431	1.4	0.52–3.79	0.505	2.03	0.75–5.44	0.161

* Odds ratios were adjusted for age and sex in unconditional logistic regression models.

† Odds ratios were adjusted for age in unconditional logistic regression models.

‡ Odds ratios were adjusted for sex in unconditional logistic regression models.

### Polygenic analysis

We obtained our results from the allele-specific and subgroup analyses by studying those SNPs polygenically. Because *MUC1* rs4072037 and *ZBTB20* rs9841504 increased gastric cancer risk in the whole population, they were used to predict the risk of gastric cancer among the Han Chinese. Those with AA-GG and AA-GC alleles had a 2.93- and 6.18-fold higher risk compared with those who had only GA-GG alleles (*P* = 0.0046†; *P* = 0.0003‡) (Table 4). Similarly, *PLCE1* rs2274223 and *PTGER4* and *PRKAA1* rs13361707 were used to predict the risk of cardia cancer in populations with a hereditary background, who faced a greater than 3-fold higher risk (*P* < 0.05*) (Table 5). More interestingly, whereas *MUC1* rs4072037, *ZBTB20* rs9841504, and *TYMS* rs2790 were suggested to improve the risk of noncardia gastric cancer (mainly the diffuse type) by 5- to 8-fold (*P* < 0.05*) (Table 6).

**Table 4:** MUC1 rs4072037 and ZBTB20 rs9841504 predict the risk of gastric cancer in all populations

*MUC1* -*ZBTB20*	Cases *N* (%)	Controls *N* (%)	Cases*vs*. Controls	
			OR	*P* value
GA-GG	12 (10.3%)	29 (28.4%)	1 (reference)	
GA-GC	7 (6.0%)	5 (4.9%)	3.38	0.0648
AA-GG	69 (59.5%)	57 (55.9%)	2.93	†0.0046
AA-GC	23 (19.8%)	9 (8.8%)	6.18	‡0.0003

† *P* < 0.01;

‡ *P* < 0.001

**Table 5:** PLCE1 rs2274223 and PTGER4 and PRKAA1 rs13361707 predict the risk of cardia cancer in populations with a hereditary background

*PLCE1* –*PRKAA1*	Cases^A^ *N* (%)	Cases^B^ *N* (%)	Controls *N* (%)	Cases^A^ *vs*. Controls		Cases^A^ *vs*. Cases^B^	
				OR	*P* value	OR	*P* value
AA-AA	7 (10.8%)	11 (21.6%)	18 (17.6%)	1 (reference)		1 (reference)	
AA-GA	19 (29.2%)	17 (33.3%)	33 (32.3%)	1.48	0.4582	1.76	0.3356
AG-AA	6 (9.2%)	2 (3.9%)	7 (6.9%)	2.20	0.2631	4.71	0.0892
AG-GA	19 (29.2%)	4 (7.8%)	16 (15.7%)	3.05	0.0428*	7.46	0.0039†

* *P* < 0.05;

† *P* < 0.01;

‡ *P* < 0.001

**Table 6:** MUC1 rs4072037, ZBTB20 rs9841504, and TYMS rs2790 predict the risk of noncardia gastric cancer (Mainly the diffuse type)

*MUC1*-*ZBTB20*-*TYMS*	Cases *N* (%)	Controls *N* (%)	Cases*vs*. Controls	
			OR	*P* value
GA-GG-AA	3 (2.6%)	9 (8.8%)	1 (reference)	
AA-GG-AA	24 (20.7%)	26 (25.5%)	2.77	0.1490
AA-GG-AG	36 (31.0%)	22 (21.6%)	4.91	0.0186*
AA-GC-AA	9 (7.8%)	4 (3.9%)	6.75	0.0270*
AA-GC-AG	11 (9.5%)	4 (3.9%)	8.25	0.0125*

* *P* < 0.05

## DISCUSSION

Our studies explored the field of hereditary gastric carcinoma in a polygenic way, and the multiplicative model showed the importance of genetic variants in early-onset gastric cancer susceptibility. By distinguishing high-risk from low-risk populations, we hoped to develop more economical and efficient screening programs, especially in developing countries with many different populations.

In the West, hereditary gastric cancer was first related to the CDH1 mutation. Since then, according to the guidelines of Oliveira and colleagues,[31] it has been suggested that diffuse familial gastric cancer or hereditary diffuse gastric cancer is similar to gastric cancer due to the CDH1 mutation. Because few families with the CDH1 mutation have been reported in Asian countries, less attention was paid to possible hereditary factors there than to environment factors (such as *Helicobacter pylori* infection and personal lifestyle). However, different incidence rates of gastric cancer were observed under the same personal and environment conditions, suggesting that low-penetrance genes other than CDH1 might play a role in gastric cancer susceptibility.

A number of genetic loci for gastric cancer susceptibility—such as *MUC1* rs4072037, *ZBTB20* rs9841504, *PSCA* rs2294008, *PLCE1* rs2274223, and *PTGER4* and *PRKAA1* rs13361707—were recently discovered by GWAS.[19–21] In order to avoid false-positive results, a large number of confirmation studies and meta-analyses followed.[22, 23, 25, 32, 33] However, the results have not always been consistent. Two crucial factors should be taken into account. First, populations of different ethnicities were enrolled and compared, which would be worthless for risk prediction. The Human Genome Project pointed out that the major/minor allele of the same SNP varied greatly in percentage among such populations. Because the baselines are not consistent across different populations, the role of each SNP in risk prediction should not be equally weighted. Second, the assignment of a variety of weights would induce different ages of onset when the existence of these SNPs was discovered. Because the loci used in our calculations were selected from previous studies and the percentages were consistent with the result in the Human Genome Project, the supportive evidence is strong.

Although many studies related to gastric cancer polymorphisms have recently been published, the application of their results in clinical and preventive medicine remains to be explored. The reasons for this are complicated. Instead of affecting protein function in carcinogenesis directly, genes such as *PSCA* may inhibit the growth of differentiated epithelial cells [21]; furthermore, SNP studies are often on the genetic level, which makes the relationship between a single molecule and changes in the stomach difficult to explain. Also, studies have shown large variations with the same SNP because of varying genetic backgrounds (e.g., involving ethnicity and gender). Our study showed that males were more susceptible to gastric cancer than females. Thus a better way to apply our findings clinically would be to classify each population in terms of the predicted percentile risk for individuals within that population.

We assumed a 40% reduction of gastric cancer risk with the gastroscopy examination, but it is not that simple. With our assumption, a greater number of loci predicting the risk of gastric cancer will be discovered. The risk estimation, however, still suggests an accurate calculation. The true benefit is a complex interaction between absolute risk and the gastroscopy operator. In China, public awareness of gastric cancer in high-risk populations should be promoted. Besides, professional education would be necessary for accepting the concept of a multiplicative genetic model. If populations at varying risk were grouped, a special screening process (e.g., gastroscopy, abdominal CT, and/or PET-CT) to be administered at given intervals could be designed for different groups.

However, we must still face the fact that most of the risk factors for gastric carcinoma are yet be discovered. At least 2 different methods need to be improved. First, technological improvements in the detection of SNPs, with both higher sensitivity and specificity, will enable more reliable predictions. Importantly, a large sample size is indeed necessary, not only for confirmation but for new findings as well.

With the discovery of more susceptible loci of gastric cancer in the future, our understanding of hereditary polygenic gastric cancer will become more complete. Disease prediction and prevention will enter a new era—the genetic era.

## MATERIALS AND METHODS

### Inclusion criteria for study subjects

Data on the characteristics of study subjects (e.g., age, sex, family history, etc.) were collected from the medical record. Histology was confirmed on the basis of biopsy specimens in the Department of Pathology at the same hospital. The gastric carcinomas were all adenocarcinomas. In this study, the inclusion criterion for diffuse-type gastric cancer was Lauren's diffuse type with poorly differentiated or signet-ring cell histology in the World Health Organization (WHO) classification or linitis plastica. The inclusion criterion for intestinal-type gastric cancer was Lauren's intestinal type with papillary, well-differentiated, or moderately differentiated histology by the WHO classification. The study was approved by the Ethics Committee of Peking University Cancer Hospital and informed consent was obtained from all subjects.

### Genotyping

Genomic DNA was extracted from venous blood with the QIAamp Blood Kit (Qiagen, Hilden, Germany) according to the manufacturer's instructions and stored at −20oC for future use. The polymerase chain reaction (PCR) was used to perform the genotyping.[34] PCR was conducted on the GeneAmp PCR System 9700 Thermal Cycler (Applied Biosystems, Foster City, CA, USA); it has a total volume of 20 μL containing 2 μL genomic DNA (around 40 ng/μL), 2 μL 10x LA PCR buffer, 0.5 μL 10 μM each primer, 2 μL 10 mmol/L dNTP, 0.2 μL Taq DNA polymerase (DRR200A, TAKARA), and 13.3 μL ddH2O. The cycling parameters were 94°C for 5 minutes, 35 cycles at 94°C for 30 seconds, 57 to 62°C (depending on the primers) for 45 seconds, 72°C for 20 seconds, and a final extension step at 72°C for 7 minutes. The PCR products were determined by 2% agarose gel electrophoresis and sequenced by an Invitrogen 3730XL genetic analyzer. The sequencing results were analyzed with Chromas software under the condition of signal/noise > 98%.

### Statistical analysis

Statistical analysis was performed using the STATA 13 software package (StataCorp LP, College Station, Texas, USA). The Hardy-Weinberg equation was used to compare the observed and expected genotype frequencies. The genotype distributions were compared with two-sided contingency tables using the χ2 test. The odds ratio (OR) and 95% confidence interval (CI) were calculated using an unconditional logistical regression model. The P value was considered significant at less than 5%.

## SUPPLEMENTARY MATERIAL TABLES



## References

[1] Jemal A, Bray F, Center MM, Ferlay J, Ward E, Forman D (2011). Global cancer statistics. CA Cancer J Clin.

[2] Ferlay J, Shin HR, Bray F, Forman D, Mathers C, Parkin DM (2010). Estimates of worldwide burden of cancer in 2008: GLOBOCAN 2008. Int J Cancer.

[3] Malfertheiner P, Venerito M, Selgrad M (2013). Helicobacter pylori infection: selected aspects in clinical management. Curr Opin Gastroenterol.

[4] Ferro A, Peleteiro B, Malvezzi M, Bosetti C, Bertuccio P, Levi F, Negri E, La Vecchia C, Lunet N (2014). Worldwide trends in gastric cancer mortality (1980-2011), with predictions to 2015, and incidence by subtype. Eur J Cancer.

[5] Bertuccio P, Chatenoud L, Levi F, Praud D, Ferlay J, Negri E, Malvezzi M, La Vecchia C (2009). Recent patterns in gastric cancer: a global overview. Int J Cancer.

[6] Anderson WF, Camargo MC, Fraumeni JF, Correa P, Rosenberg PS, Rabkin CS (2010). Age-specific trends in incidence of noncardia gastric cancer in US adults. JAMA.

[7] National Cancer Center of China (2007). China Cancer Incidence and Death (1988-2002) (Chinese). Chinese Medicine and Technology Publisher.

[8] Liao LM, Hofmann JN, Kamangar F, Strickland PT, Ji BT, Yang G, Li HL, Rothman N, Zheng W, Chow WH, Gao YT, Shu XO (2014). Polycyclic aromatic hydrocarbons and risk of gastric cancer in the Shanghai Women's Health Study. Int J Mol Epidemiol Genet.

[9] Chiu HF, Tsai SS, Chen PS, Liao YH, Liou SH, Wu TN, Yang CY (2011). Traffic air pollution and risk of death from gastric cancer in Taiwan: petrol station density as an indicator of air pollutant exposure. J Toxicol Environ Health A.

[10] Sitarz R, Leguit RJ, de Leng WW, Morsink FH, Polkowski WP, Maciejewski R, Offerhaus GJ, Milne AN (2009). Cyclooxygenase-2 mediated regulation of E-cadherin occurs in conventional but not early-onset gastric cancer cell lines. Cell Oncol.

[11] Milne AN, Offerhaus GJ (2010). Early-onset gastric cancer: Learning lessons from the young. World J Gastrointest Oncol.

[12] Shiovitz S, Korde LA (2015). Genetics of Breast Cancer: A Topic in Evolution. Ann Oncol.

[13] Pinheiro H, Oliveira C, Seruca R, Carneiro F (2014). Hereditary diffuse gastric cancer - pathophysiology and clinical management. Best Pract Res Clin Gastroenterol.

[14] Zhu ZG, Yu YY, Zhang Y, Ji J, Zhang J, Liu BY, Chen XH, Lu Y, Jiang HS, Bu L, Hu LD, Kong XY (2004). Germline mutational analysis of CDH1 and pathologic features in familial cancer syndrome with diffuse gastric cancer/breast cancer proband in a Chinese family. Eur J Surg Oncol.

[15] Kim S, Chung JW, Jeong TD, Park YS, Lee JH, Ahn JY, Kim do H, Choi KD, Lee W, Song HJ, Lee GH, Chun S, Jung HY (2013). Searching for E-cadherin gene mutations in early onset diffuse gastric cancer and hereditary diffuse gastric cancer in Korean patients. Fam Cancer.

[16] Yamada M, Fukagawa T, Nakajima T, Asada K, Sekine S, Yamashita S, Okochi-Takada E, Taniguchi H, Kushima R, Oda I, Saito Y, Ushijima T, Katai H (2014). Hereditary diffuse gastric cancer in a Japanese family with a large deletion involving CDH1. Gastric Cancer.

[17] Wang GY, Lu CQ, Zhang RM, Hu XH, Luo ZW (2008). The E-cadherin gene polymorphism 160C- > A and cancer risk: A HuGE review and meta-analysis of 26 case-control studies. Am J Epidemiol.

[18] Wang L, Wang G, Lu C, Feng B, Kang J (2012). Contribution of the −160C/A polymorphism in the E-cadherin promoter to cancer risk: a meta-analysis of 47 case-control studies. PLoS One.

[19] Shi Y, Hu Z, Wu C, Dai J, Li H, Dong J, Wang M, Miao X, Zhou Y, Lu F, Zhang H, Hu L, Jiang Y (2011). A genome-wide association study identifies new susceptibility loci for non-cardia gastric cancer at 3q13 31 and 5p13.1. Nat Genet.

[20] Abnet CC, Freedman ND, Hu N, Wang Z, Yu K, Shu XO, Yuan JM, Zheng W, Dawsey SM, Dong LM, Lee MP, Ding T, Qiao YL (2010). A shared susceptibility locus in PLCE1 at 10q23 for gastric adenocarcinoma and esophageal squamous cell carcinoma. Nat Genet.

[21] Sakamoto H, Yoshimura K, Saeki N, Katai H, Shimoda T, Matsuno Y, Saito D, Sugimura H, Tanioka F, Kato S, Matsukura N, Matsuda N, Study Group of Millennium Genome Project for Cancer (2008). Genetic variation in PSCA is associated with susceptibility to diffuse-type gastric cancer. Nat Genet.

[22] Umar M, Upadhyay R, Mittal B (2013). PLCE1 rs2274223 A > G polymorphism and cancer risk: a meta-analysis. Tumour Biol.

[23] Mocellin S, Verdi D, Pooley KA, Nitti D (2015). Genetic variation and gastric cancer risk: a field synopsis and meta-analysis. Gut.

[24] Gu X, Zhang W, Xu L, Cai D (2014). Quantitative assessment of the influence of prostate stem cell antigen polymorphisms on gastric cancer risk. Tumour Biol.

[25] Liu X, Wang Z, Zhang X, Chang J, Tang W, Gan L, Wu Z, Li J (2014). MUC1 gene polymorphism rs4072037 and susceptibility to gastric cancer: a meta-analysis. Springerplus.

[26] Shen R, Liu H, Wen J, Liu Z, Wang LE, Wang Q, Tan D, Ajani JA, Wei Q (2015). Genetic polymorphisms in the microRNA binding-sites of the thymidylate synthase gene predict risk and survival in gastric cancer. Mol Carcinog.

[27] Pharoah PD, Antoniou AC, Easton DF, Ponder BA (2008). Polygenes, risk prediction, and targeted prevention of breast cancer. N Engl J Med.

[28] Wang P, Ye D, Guo J, Liu F, Jiang H, Gong J, Gu C, Shao Q, Sun J, Zheng SL, Yu H, Lin X, Xia G (2014). Genetic score of multiple risk-associated single nucleotide polymorphisms is a marker for genetic susceptibility to bladder cancer. Genes Chromosomes Cancer.

[29] Lu Y, Ek WE, Whiteman D, Vaughan TL, Spurdle AB, Easton DF, Pharoah PD, Thompson DJ, Dunning AM, Hayward NK, Chenevix-Trench G, Macgregor S, Q-MEGA and AMFS Investigators; ANECS-SEARCH; UKOPS-SEARCH; BEACON Consortium (2014). Most common ‘sporadic’ cancers have a significant germline genetic component. Hum Mol Genet.

[30] Pharoah PD, Antoniou A, Bobrow M, Zimmern RL, Easton DF, Ponder BA (2002). Polygenic susceptibility to breast cancer and implications for prevention. Nat Genet.

[31] Oliveira C, Seruca R, Hoogerbrugge N, Ligtenberg M, Carneiro F (2013). Clinical utility gene card for: Hereditary diffuse gastric cancer (HDGC). Eur J Hum Genet.

[32] Dai N, Zheng M, Wang C, Ji Y, Du J, Zhu C, He Y, Zhu M, Zhu X, Sun M, Dai J, Ma H, Chen J (2014). Genetic variants at 8q24 are associated with risk of esophageal squamous cell carcinoma in a Chinese population. Cancer Sci.

[33] Duan X, Li X, Lou H, Geng T, Jin T, Liang P, Li S, Long Y, Chen C (2014). Genetic association of PLCE1, C11orf92-C11orf93, and NOC3L with colorectal cancer risk in the Han population. Tumour Biol.

[34] Gao J, Dang Y, Sun N, Li J, Shen L (2012). C-KIT mutations were closely associated with the response to Imatinib in Chinese advanced gastrointestinal stromal tumor patients. Med Oncol.

