# Supplementary material for: Risk prediction for early-onset gastric carcinoma: a case-control study of polygenic gastric cancer in Han Chinese with hereditary background
Source: Oncotarget. 2016 Apr 26;7(23):33608–15. doi: 10.18632/oncotarget.9025 (PMC5085106; doi:10.18632/oncotarget.9025)
Supplement: Supplementary file 1 [file oncotarget-07-33608-s001.pdf]

Risk prediction for early-onset gastric carcinoma: a case-control study of polygenic gastric cancer in Han Chinese with hereditary background

Supplementary Material

Supplementary Methods

The multiplicative polygenic model

Each single-nucleotide polymorphism (SNP) has at least two alleles in two loci, which are named high-risk and low-risk alleles in genetic risk studies. Two copies of each locus combine three possibilities: two low-risk alleles, one low-risk and one high-risk allele, or two high-risk alleles. Three GWASs reported five susceptibility loci of gastric cancer globally [1-3]. In addition to rs2790 in the allele-specific study [4], we analysed 6 SNPs in our study. Assuming each locus is independent without interactions and the risk is allele-dose-dependent with a simple multiplicative (log-additive) effect, we calculated the combined effect on the relative-risk scale. In consistence with the polygenic model, the multiplicative model is adequate for cancer susceptibility study [5].

We assume the relative risk in monozygotic twins ($\lambda_{monozygotic}$) and siblings ($\lambda_{sibling}$) are related to each other and an estimation of $\lambda_{sibling}$ equal to 2, as observed from many epidemiologic studies [6-8], which concludes

$$\lambda_{monozygotic} = \lambda_{sibling}{}^{2} = 4$$

Assuming the standard deviation (s.d.) of monozygotic twins is in the polygenic log-normal risk distribution:

$$\mathrm{s.d.} = \sqrt{\ln\left(\lambda_{monozygotic}\right)}$$

this equation solves to predict an s.d. of 1.2.

According to the deduction in multiplicative polygenic model of breast cancer [9], the population attributable fraction is given by

$$\frac{2p(1-p)(R-1)}{2p(1-p)(R-1)+1} + \frac{(1-p)^2(R^2-1)}{(1-p)^2(R^2-1)+1}$$

Where p is the frequency and R is the relative risk for a single allele i.

Besides, r is defined as the log risk, and E as the expected value of r.

For the single allele i, the variance V_i of the risk distribution is given by

$$V_i = (1-p)^2E^2 + 2p(1-p)(r-E)^2 + p^2(2r-E)^2$$

For multiple risk alleles, the risk distribution tends towards the normal with variance

$$V = \sum V_i$$

where V is the total genetic variance with an estimation value of 1.44.

Thus, the risk fraction explained by a single allele is estimated as V_i/V

Supplementary Table 1 Hardy–Weinberg equation comparison between the observed and expected frequencies of common gastric-cancer susceptibility alleles.

**dbSNP No.**	**Gene**	**Pearson chi^2**	***p*-value**	**LR chi^2**	***p*-value**	**Exact Significance**
rs4072037	*MUC1*	0.92	0.34	0.99	0.32	0.43
rs9841504	*ZBTB20*	0.27	0.60	0.26	0.61	0.66
rs2294008	*PSCA*	1.38	0.24	1.36	0.24	0.27
rs2274223	*PLCE1*	1.12	0.29	1.09	0.30	0.32
rs13361707	*PTGER4 and PRKAA1*	1.84	0.17	1.85	0.17	0.21
rs2790	*TYMS*	0.34	0.56	0.34	0.56	0.61

Supplementary Table 2 The frequency of each risk allele in cases and controls.

dbSNP No.	Gene	risk allele	frequency (%)				*HGP (%)	
			Cases[A]	Cases[B]	Cases	Controls	*CHB	*CHS
rs4072037	*MUC1*	A	89.2	89.2	89.2	82.4	83.5	81.0
rs9841504	*ZBTB20*	C	16.9	15.7	16.4	8.3	10.7	13.8
rs2294008	*PSCA*	T	40.0	26.5	34.1	28.4	24.8	27.1
rs2274223	*PLCE1*	G	28.5	14.7	22.4	20.6	18.9	21.9
rs13361707	*PTGER4 and PRKAA1*	G	54.6	48.0	51.7	51.0	49.5	42.4
rs2790	*TYMS*	G	43.1	34.3	39.2	34.8	41.3	38.1

*HGP: Human Genome Project; CHB: Chinese Han Beijing; CHS: Chinese Han Southern.

Supplementary Table 3 Comparison of each SNP between Cases[A] and Controls (Cases[B]) in the co-dominant, dominant and recessive models.

(Cases[A] vs.Controls)	#Co-dominant model						#Dominant model			#Recessive model		
	*Mm vs. MM			*mm vs. MM								
dbSNP No.	OR	95% CI	*p*-value	OR	95% CI	*p*-value	OR	95% CI	*p*-value	OR	95% CI	*p*-value
rs4072037	0.49	0.23-1.02	0.055	/	/	0.995	0.47	0.23-0.99	0.046	/	/	0.999
rs9841504	2.13	0.97-4.66	0.059	3.84	0.32-41.66	0.299	2.22	1.04-4.75	0.039	3.12	0.27-35.50	0.359
rs2294008	1.65	0.84-3.24	0.147	2.97	1.02-8.62	0.045	1.85	0.97-3.52	0.061	2.29	0.84-6.24	0.104
rs2274223	2.03	1.04-3.96	0.037	1.38	0.36-5.28	0.635	1.92	1.02-3.64	0.044	1.05	0.28-3.88	0.944
rs13361707	1.77	0.75-4.19	0.193	1.66	0.61-4.49	0.322	0.94	0.45-1.95	0.863	0.58	0.25-1.32	0.193
rs2790	1.90	0.94-3.81	0.073	1.77	0.66-4.77	0.257	1.87	0.96-3.64	0.066	1.23	0.50-3.00	0.655
(Cases[A] vs. Cases[B])	#Co-dominant model						#Dominant model			#Recessive model		
	*Mm vs. MM			*mm vs. MM								
dbSNP No.	OR	95% CI	*p*-value	OR	95% CI	*p*-value	OR	95% CI	*p*-value	OR	95% CI	*p*-value
rs4072037	0.82	0.25-2.70	0.742	/	/	0.993	0.64	0.20-2.00	0.443	/	/	0.996
rs9841504	0.54	0.20-1.44	0.217	1.36	0.07-27.28	0.842	0.57	0.22-1.47	0.242	1.60	0.08-31.77	0.758
rs2294008	2.64	1.05-6.67	0.040	2.62	0.77-8.92	0.123	2.61	1.13-6.03	0.024	1.77	0.55-5.64	0.335
rs2274223	4.25	1.64-11.03	0.003	1.07	0.08-14.05	0.961	3.80	1.51-9.55	0.004	0.59	0.05-7.30	0.678
rs13361707	5.28	1.53-18.25	0.009	3.98	1.01-15.65	0.048	0.90	0.36-2.27	0.823	0.21	0.06-0.68	0.010
rs2790	2.37	0.96-5.83	0.061	2.15	0.58-7.97	0.252	2.32	0.98-5.52	0.057	1.27	0.39-4.15	0.689

* M represents the major allele and m represents the minor allele in each SNP.

# Co-dominant model compares the difference between Mm, mm and MM; Dominant model compares the difference between (Mm+mm) and MM; Recessive model compares the difference between mm and (MM+Mm). All models are adjusted for sex, age, location and pathology in logistic regression model.

Supplementary Table 4 Population attributable risk and total variance in risk.

dbSNP No.	Gene*	Fraction of Total Variance in Risk Explained § %	Population Attributable Risk §
rs4072037	*MUC1*	6.7	78.0
rs9841504	*ZBTB20*	7.0	18.8
rs2294008	*PSCA*	8.7	37.2
rs2274223	*PLCE1*	4.4	20.9
rs13361707	*PTGER4 and PRKAA1*	1.2	19.3
rs2790	*TYMS*	4.0	27.6

* These genes are within the linkage-disequilibrium block or blocks defined by the associated variant and are plausible candidates for the causal gene.

§ See the Supplementary Methods for details.

Supplementary Table 5 Subgroup analysis between Cases[A] and Cases[B]*.

Exposure		MUC1 rs4072037			ZBTB20 rs9841504			PSCA rs2294008		
		OR	95% CI	*p*-value	OR	95% CI	*p*-value	OR	95% CI	*p*-value
Sex										
	Male	1.12	0.30-4.20	0.87	0.82	0.26-2.62	0.74	1.84	0.83-4.08	0.132
	Female	3.14	0.55-17.81	0.196	0.53	0.15-1.93	0.338	2.18	0.84-5.72	0.111
Age										
	<=40	0.9	0.23-3.56	0.884	1.07	0.30-3.77	0.919	3.47	1.20-10.01	0.021
	40-50	3.56	0.66-19.27	0.141	0.73	0.23-2.29	0.586	1.81	0.82-3.99	0.14
Location										
	Non-cardia	1.16	0.39-3.45	0.791	0.56	0.22-1.39	0.211	2.47	1.20-5.06	0.014
	Cardia	/	/	0.996	/	/	0.995	1.88	0.45-7.96	0.389
Pathology										
	Diffuse	3.67	0.68-19.99	0.132	0.58	0.20-1.69	0.318	2.24	0.92-5.46	0.076
	Intestinal	2.45	0.35-17.03	0.364	0.96	0.06-15.82	0.977	3.35	0.98-11.42	0.053
	Mixed	/	/	0.997	5.33	0.43-66.34	0.193	0.18	0.02-1.37	0.097

Exposure		PLCE1 rs2274223			PTGER4 and PRKAA1 rs13361707			TYMS rs2790		
		OR	95% CI	*p*-value	OR	95% CI	*p*-value	OR	95% CI	*p*-value
Sex										
	Male	5.37	1.83-15.78	0.002	1.6	0.75-3.40	0.222	2.36	1.09-5.13	0.029
	Female	1.07	0.31-3.71	0.919	1.69	0.65-4.39	0.281	0.88	0.34-2.26	0.794
Age										
	<=40	0.95	0.30-3.04	0.928	1.82	0.66-5.01	0.245	1.4	0.52-3.76	0.504
	40-50	7.42	2.23-24.74	0.001	1.55	0.74-3.22	0.242	2.06	0.96-4.44	0.063
Location										
	Non-cardia	3.02	1.14-8.04	0.027	1.42	0.72-2.79	0.313	1.77	0.89-3.53	0.104
	Cardia	2.84	0.72-11.17	0.135	2.7	0.75-9.69	0.129	1.6	0.47-5.51	0.456
Pathology										
	Diffuse	4.4	1.38-14.02	0.012	1.07	0.47-2.43	0.874	1.44	0.65-3.22	0.371
	Intestinal	3.56	0.82-15.50	0.091	1.93	0.60-6.20	0.269	1.38	0.43-4.46	0.589
	Mixed	0.18	0.01-3.61	0.26	2.73	0.44-16.81	0.279	3.77	0.60-23.63	0.156

* Odds ratios were adjusted for age, sex, location, and pathology in unconditional logistic regression models.

References

1. Shi Y, Hu Z, Wu C, Dai J, Li H, et al. (2011) A genome-wide association study identifies new susceptibility loci for non-cardia gastric cancer at 3q13.31 and 5p13.1. Nat Genet 43: 1215-1218.

2. Abnet CC, Freedman ND, Hu N, Wang Z, Yu K, et al. (2010) A shared susceptibility locus in PLCE1 at 10q23 for gastric adenocarcinoma and esophageal squamous cell carcinoma. Nat Genet 42: 764-767.

3. Sakamoto H, Yoshimura K, Saeki N, Katai H, Shimoda T, et al. (2008) Genetic variation in PSCA is associated with susceptibility to diffuse-type gastric cancer. Nat Genet 40: 730-740.

4. Shen R, Liu H, Wen J, Liu Z, Wang LE, et al. (2014) Genetic polymorphisms in the microRNA binding-sites of the thymidylate synthase gene predict risk and survival in gastric cancer. Mol Carcinog.

5. Pharoah PD, Antoniou A, Bobrow M, Zimmern RL, Easton DF, et al. (2002) Polygenic susceptibility to breast cancer and implications for prevention. Nat Genet 31: 33-36.

6. Gao Y, Hu N, Han X, Giffen C, Ding T, et al. (2009) Family history of cancer and risk for esophageal and gastric cancer in Shanxi, China. BMC Cancer 9: 269.

7. Jiang X, Tseng CC, Bernstein L, Wu AH (2014) Family history of cancer and gastroesophageal disorders and risk of esophageal and gastric adenocarcinomas: a case-control study. BMC Cancer 14: 60.

8. Yatsuya H, Toyoshima H, Mizoue T, Kondo T, Tamakoshi K, et al. (2002) Family history and the risk of stomach cancer death in Japan: differences by age and gender. Int J Cancer 97: 688-694.

9. Pharoah PD, Antoniou AC, Easton DF, Ponder BA (2008) Polygenes, risk prediction, and targeted prevention of breast cancer. N Engl J Med 358: 2796-2803.
